# Hypothermia on the first day of ICU admission leads to increased in-hospital mortality in patients with subarachnoid hemorrhage

**DOI:** 10.1038/s41598-024-60657-8

**Published:** 2024-04-28

**Authors:** Wenyuan Du, Jingmian Yang, Yanfang Lou, Jiahua You

**Affiliations:** Department of Neurology, Shijiazhuang Traditional Chinese Medicine Hospital, Shijiazhuang, Hebei China

**Keywords:** Stroke, Stroke

## Abstract

The relationship between early spontaneous hypothermia and adverse clinical outcomes in patients with subarachnoid hemorrhage (SAH) has not been paid much attention. We designed this retrospective cohort study to determine this relationship by analyzing the association between the lowest body temperature (T-lowest) on the first day of ICU admission and in-hospital mortality. In this study, 550 participants with non-traumatic SAH were chosen from the Medical Information Mart for Intensive Care (MIMIC)-IV database. Multivariate Cox regression analysis showed that T-lowest was nonlinearity correlated with in-hospital mortality (HR = 0.72, 95% CI: 0.59–0.86, *p* < 0.001). We divided the T-lowest into quartile groups. In comparison to reference group Q1 (31.30–36.06 ℃), group Q3 (36.56–36.72 ℃) had a 50% lower risk of death in the hospital (HR: 0.5, 95% CI: 0.28–0.87, *p* = 0.014). We further confirmed the curve-like relationship between T-lowest and in-hospital mortality using restricted cubic splines. The mortality is lowest when the T-lowest is close to 36.5 °C, and the risk of death is increased when the temperature is lower or higher than that. Our study demonstrates that in-hospital mortality is associated with T-lowest. Patients with non-traumatic SAH are at increased risk of death if their body temperature on the first day of ICU admission is too low.

## Introduction

Subarachnoid hemorrhage (SAH) is a serious disease with high mortality and disability^1^. Non-traumatic SAH occurs with an incidence of (7.2–9.0) per 100,000 people per year^2^ and is a common type of stroke in intensive care units (ICU). Even if the patients survive, they are still prone to residual neurological impairment, which seriously affects their basic life. Many clinical trials have shown that early, active, and reasonable treatment can improve the clinical outcome of SAH patients^3–5^. For patients with severe SAH, neurological intensive care is required due to primary brain tissue damage, secondary brain tissue ischemia, increased intracranial pressure, and systemic complications. The level of neurocritical management is directly related to the prognosis of the disease^6^.

In ICU management of SAH patients, the management of body temperature has attracted great attention, although the effects of body temperature on the brain and the optimal treatment are still inconclusive^7,8^. It is well known that 41–72% of patients develop fever after hemorrhage^9–11^. Hyperthermia results from infection on the one hand and neurogenic mechanisms on the other^6^. Numerous studies have shown that hyperthermia can lead to increased brain energy metabolism, cerebral congestion, intracranial hypertension, destruction of the blood–brain barrier, cerebral edema, excitatory toxicity, and other effects^12–14^. Hyperpyrexia was independently associated with prolonged ICU stay and poor prognosis after SAH^15–17^. In contrast, 166 patients with aneurysmal SAH were included in a recent observational study, and the results indicated that individuals with SAH seemed to have a biphasic body temperature pattern. Early in the disease (one to three days after beginning), the majority of patients exhibit normothermia, and in certain cases, they also exhibit spontaneous hypothermia. A period of 4–10 days of vasospasm with an increase in body temperature follows. Early after the incident, there was a spontaneous decrease in mean body temperature in individuals with a higher Fisher rating, more indicators of brain injury, and a worse prognosis for the future^18^. In this study, a better prognosis was in fact linked to an early increase in mean temperature of the body, and a worse prognosis was linked to an increased mean temperature in vasospasm; however, the multiple regression models did not clearly show these connections. Two other literatures have also reported spontaneous hypothermia in patients with SAH in the early stage of onset (within 4 h)^19,20^, indicating that the fall in temperature of the body is not a result of circadian rhythm and has nothing to do with where the brain aneurysm is located. It is speculated that early hypothermia in SAH patients may be an internal neural protection mechanism or a change in temperature setpoint caused by brain stem/hypothalamus dysfunction. No additional studies have been found on the relationship between early hypothermia and clinical outcomes for SAH individuals.

In individuals with SAH, there is a complicated correlation between body temperature and clinical results. Although there are currently some reports on temperature studies in SAH patients, there is still a lack of literature on the relationship between temperature at early admission and prognosis. Investigating this relationship will be useful for early prognosis assessment and treatment intervention for SAH patients. We examined the correlation between in-hospital mortality for patients with non-traumatic SAH and the lowest body temperature on the first day of ICU admission using data from the MIMIC-IV database.

## Materials and methods

### Data sources

The big public database MIMIC-IV^21^ allows free downloads of the study’s data. The Beth Israel Deaconess Medical Center (BIDMC) patients who were admitted were listed in the database between 2008 and 2019. The first author was permitted to access the database (Record ID: 56305796) after passing the Protecting Human Research Participants exam and the National Institutes of Health (NIH) training course. Data collection does not need informed permission because all information is anonymized to safeguard patient privacy. The STROBE statement^22^ is followed by this study.

### Study population

A total of 559 non-traumatic SAH patients were admitted to the ICU, according to ICD-9 code 430 and ICD-10 codes I60, I600-I609, I6000-I6002, I6010-I6012, I6020-I6022, I6030-I6032, and I6050-I6052^23^. The patients who satisfied the requirements were chosen for analysis: First ICU hospitalization; older than 18 years of age. Participants lacking body temperature on the first day of ICU admission would be excluded. Finally, the research comprised 550 participants.

### Data extraction

Using PostgreSQL, the following variables were retrieved for this study: (i) Sex, age, and race were among the demographic factors; (ii) Respiratory rate (RR), heart rate, mean arterial blood pressure (MBP), blood oxygen saturation (SpO_2_), the lowest body temperature (T-lowest), and mean body temperature (T-mean) on the first day of ICU admission; (iii) The Charlson comorbidity index, chronic lung illness, paraplegia, hypertension, diabetes, sepsis, renal disease, cancer, severe liver disease, and congestive heart failure are among the complications; (iv) Laboratory variables included baseline blood glucose, platelets, white blood cell (WBC) count, red blood cells (RBC), calcium, sodium, hemoglobin, PT, APTT, creatinine, and urea nitrogen tested for the first time within a 24 h period following ICU admission; (v) Baseline Glasgow Coma Score (GCS), Acute Physiology III (APSIII) score, SOFA score; (vi) Hydrocephalus and in-hospital mortality (endpoints).

### Statistical analysis

Continuous variables are represented using the formula mean ± standard deviation (SD) or median (quartile 1, quartile 3). Either the Mann–Whitney *U*-test or the t-test is used, depending on whether the distribution is normal. The categorical variable is given as the percentage of instances, and the Chi-square test (or Fisher’s exact method) is performed to examine the variations among the T-lowest groups (grouped by quartile).

Cox proportional hazard regression analysis, both univariate and multivariate, was employed. Based on the following criteria, confounding variables were screened: (i) Considering the multicollinearity problem between covariables; (ii)Univariate analysis results; (iii) There are at least 10 valid sample sizes for each variable factor. In this study, the number of in-hospital non-survival cases was 129, and we believe that the number of adjustable variables ≤ 12 is reasonable. In the results of univariate analysis (Supplementary Table 3), 19 variables were associated with in-hospital mortality (*p* < 0.05), including age, ethnicity, heart rate, RR, SpO_2_, blood glucose, WBC, Charlson comorbidity index, Cr, endovascular therapy, GCS, Severe liver disease, Hemoglobin, Platelets, Calcium, PT, BUN, APSIII, and SOFA. Under the consideration of statistical efficacy, variables with significant statistical differences (*p* < 0.01) were preferentially selected for inclusion in the Model, and Severe liver disease (*p* = 0.037), Hemoglobin (*p* = 0.013), Platelets (*p* = 0.01) Calcium (*p* = 0.013), SOFA (*p* = 0.013) were excluded. Since many indicators of Vital signs, the Charlson comorbidity index, and Laboratory results are included in APSIII scores, to avoid Multicollinearity, APSIII scores are not considered to be included in the adjustment Model. PT is mainly associated with severe liver disease, blood disease, and malignancy, and these items have been included in the Charlson comorbidity index. To avoid Multicollinearity, PT is not included in the Model. For clinical patients with renal failure whose Cr and BUN often have the same change trend, BUN is not included in the multivariate analysis model, also to avoid co-linearity. To sum up, considering the problems of statistical efficacy, *p*-value, and multicollinearity, we finally selected 11 variables from the results of Univariate analysis, including age, ethnicity, heart rate, RR, SpO_2_, blood glucose, WBC, Charlson comorbidity index, Cr, endovascular therapy, and GCS. Although univariate analysis showed no significant association between sex and in-hospital mortality (HR: 0.86, 95%CI: 0.61,1.23, *p* = 0.411), as an important demographic feature, has been included in the Multivariate analysis Model in other published relevant literature (although there is no correlation in univariate analysis)^23–25^, so we also take sex as one of the covariables. Therefore, a total of 12 covariates are adjusted in Model II. We adjust for sex, age, and race in Model I by referring to published papers^23–25^. Otherwise, DCI and intracranial hypertension are important variables affecting prognosis and mortality and should be collected and included in multivariate models. Unfortunately, this data is not available in the MIMIC-IV database. In addition, we carefully selected confounders from a clinical point of view and related epidemiology of non-traumatic subarachnoid hemorrhage based on the Directed Acyclic Graph (DAG) and constructed new models to confirm our findings. The new models contain 7 confounding variables, including age, GCS score, endovascular therapy, Charlson comorbidity index, sepsis, Clipping, and sex. It was explored whether there was a curve relationship between T-lowest and in-hospital mortality using restricted cubic spline analysis^26^. We conducted interaction and stratified analyses based on GCS (< 8 and ≥ 8 scores), sex, and age (< 60 and ≥ 60 years). The median or mean is used to fill in the missing numbers. Additional Table 1 gives information on the values that are missing.

**Table 1:** Population characteristics by quartiles of the lowest body temperature on the first day of ICU admission.

Variables	Quartiles of T-lowest					*p-value*
	Total (n = 550)	Q1 (31.30–36.06 ^◦^C) (n = 133)	Q2 (36.10–36.50 ^◦^C) (n = 124)	Q3 (36.56–36.72 ^◦^C) (n = 129)	Q4 (36.72–38.22 ^◦^C) (n = 164)	
Demographic						
Female, n (%)	312 (56.7)	83 (62.4)	65 (52.4)	81 (62.8)	83 (50.6)	0.068
Age, years	61.1 ± 14.7	66.5 ± 13.4	58.8 ± 14.2	58.3 ± 14.7	60.5 ± 15.3	< 0.001
Ethnicity, n (%)						
White	345 (62.7)	88 (66.2)	79 (63.7)	82 (63.6)	96 (58.5)	0.326
Black	46 (8.4)	16 (12)	9 (7.3)	9 (7)	12 (7.3)	
Asian	18 (3.3)	5 (3.8)	3 (2.4)	6 (4.7)	4 (2.4)	
Other	141 (25.6)	24 (18)	33 (26.6)	32 (24.8)	52 (31.7)	
Vital signs						
Heart rate, beats/min	78.0 ± 13.8	76.0 ± 14.1	77.4 ± 13.6	77.7 ± 14.1	80.2 ± 13.1	0.067
MBP, mmHg	81.7 ± 9.0	81.6 ± 9.8	82.2 ± 8.5	82.1 ± 9.6	81.1 ± 8.4	0.676
RR, times/min	18.0 ± 3.3	17.9 ± 3.6	17.1 ± 3.0	17.9 ± 3.1	18.7 ± 3.4	< 0.001
SpO_2_, %	97.7 (96.0, 98.9)	98.1 (96.3, 99.2)	97.6 (96.4, 98.8)	97.1 (95.8, 98.8)	97.7 (95.9, 99.1)	0.044
Comorbidities, n (%)						
Myocardial infarction	41 (7.5)	15 (11.3)	6 (4.8)	8 (6.2)	12 (7.3)	0.226
Congestive heart failure	41 (7.5)	13 (9.8)	9 (7.3)	5 (3.9)	14 (8.5)	0.294
Chronic pulmonary disease	78 (14.2)	17 (12.8)	24 (19.4)	16 (12.4)	21 (12.8)	0.317
Hypertension	276 (50.2)	74 (55.6)	60 (48.4)	56 (43.4)	86 (52.4)	0.217
Diabetes	75 (13.6)	28 (21.1)	9 (7.3)	18 (14)	20 (12.2)	0.013
Paraplegia	64 (11.6)	15 (11.3)	9 (7.3)	18 (14)	22 (13.4)	0.32
Sepsis	255 (46.4)	70 (52.6)	43 (34.7)	54 (41.9)	88 (53.7)	0.004
Renal disease	30 (5.5)	11 (8.3)	6 (4.8)	6 (4.7)	7 (4.3)	0.433
Malignant cancer	22 (4.0)	9 (6.8)	3 (2.4)	6 (4.7)	4 (2.4)	0.212
Severe liver disease	7 (1.3)	1 (0.8)	3 (2.4)	1 (0.8)	2 (1.2)	0.687
Charlson comorbidity index	4.0 (3.0, 6.0)	5.0 (4.0, 6.0)	4.0 (3.0, 6.0)	4.0 (3.0, 6.0)	4.0 (3.0, 6.0)	< 0.001
Laboratory results						
Glucose, mg/dl	142.2 ± 37.0	156.2 ± 42.7	134.9 ± 35.9	137.3 ± 35.4	140.3 ± 30.9	< 0.001
RBC, 10^12^/L	4.0 ± 0.7	3.9 ± 0.6	4.0 ± 0.7	4.1 ± 0.7	4.1 ± 0.7	0.418
Hemoglobin, g/L	11.9 ± 1.9	11.5 ± 1.9	11.9 ± 2.1	12.0 ± 1.9	12.0 ± 1.8	0.047
Platelets, 10^9^/L	216.9 ± 87.9	215.6 ± 96.4	220.7 ± 93.3	215.8 ± 89.6	215.8 ± 74.6	0.960
WBC, 10^9^/L	11.1 ± 4.9	11.2 ± 5.4	10.5 ± 3.8	11.0 ± 5.2	11.6 ± 4.8	0.322
Sodium, mmol/L	138.0 ± 3.9	138.3 ± 4.3	138.4 ± 3.4	137.7 ± 4.5	137.7 ± 3.5	0.340
Calcium, mmol/L	8.3 ± 0.7	8.2 ± 0.7	8.3 ± 0.7	8.4 ± 0.8	8.4 ± 0.6	0.251
PT, s	12.2 (11.3, 13.0)	12.4 (11.9, 13.4)	12.2 (11.3, 13.1)	11.8 (11.1, 12.7)	12.1 (11.1, 12.9)	< 0.001
APTT, s	26.2 (23.7, 28.5)	24.9 (23.3, 27.6)	26.4 (23.3, 29.2)	26.7 (24.6, 29.2)	26.4 (24.2, 28.3)	0.005
Cr (mg/dL)	0.7 (0.6, 0.9)	0.8 (0.6, 1.0)	0.7 (0.6, 0.9)	0.7 (0.6, 0.9)	0.8 (0.6, 1.0)	0.093
BUN (mg/dL)	13.0 (10.0, 17.0)	14.0 (10.0, 18.0)	12.0 (10.0, 16.0)	11.0 (9.0, 17.0)	13.0 (10.0, 16.0)	0.040
Therapy, n (%)						
Endovascular therapy of aneurysm	195 (35.5)	48 (36.1)	50 (40.3)	41 (31.8)	56 (34.1)	0.534
Clipping of aneurysm	38 ( 6.9)	9 (6.8)	7 (5.6)	9 (7)	13 (7.9)	0.902
Scores						
GCS	13.0 (7.0, 14.0)	13.0 (7.0, 14.0)	13.0 (7.0, 14.0)	13.0 (8.0, 14.0)	10.0 (6.0, 14.0)	0.135
APSIII	45.7 ± 24.0	51.1 ± 25.9	43.4 ± 22.3	42.0 ± 22.9	46.1 ± 23.9	0.011
SOFA	3.0 (2.0, 4.0)	3.0 (2.0, 4.0)	3.0 (2.0, 4.0)	2.0 (2.0, 4.0)	2.0 (2.0, 3.0)	0.539
Outcomes						
Hydrocephalus, n (%)	163 (29.6)	41 (30.8)	32 (25.8)	33 (25.6)	57 (34.8)	0.257
hospital mortality, n (%)	129 (23.5)	43 (32.3)	26 (21)	19 (14.7)	41 (25)	0.008

T-lowest, the lowest body temperature on the first day of ICU admission.

*MBP* mean blood pressure, *RR* respiratory rate, *SpO*_*2*_ percutaneous oxygen saturation, *RBC* red blood cell, *WBC* white blood cell, *PT* prothrombin time, *APTT* activated partial thromboplastin time, *Cr* creatinine, *BUN* blood urea nitrogen, *GCS*, glasgow coma score, *APSIII* *score* acute physiology III score, *SOFA* sequential organ failure assessment.

Statistical analysis was performed using packages R 3.3.2 (http:// www.R-project.org) and Free Statistics software 1.8^27,28^. Statistical significance was defined as *p* < 0.05 (bilateral test).

### Ethics statement

The Massachusetts Institute of Technology and Beth Israel Deaconess Medical Center both examined and approved the human subjects-involved investigations. Patients’ informed permission was not necessary for this study since health information was anonymized.

## Results

### Baseline characteristics of the research participants

**Figure 1:**
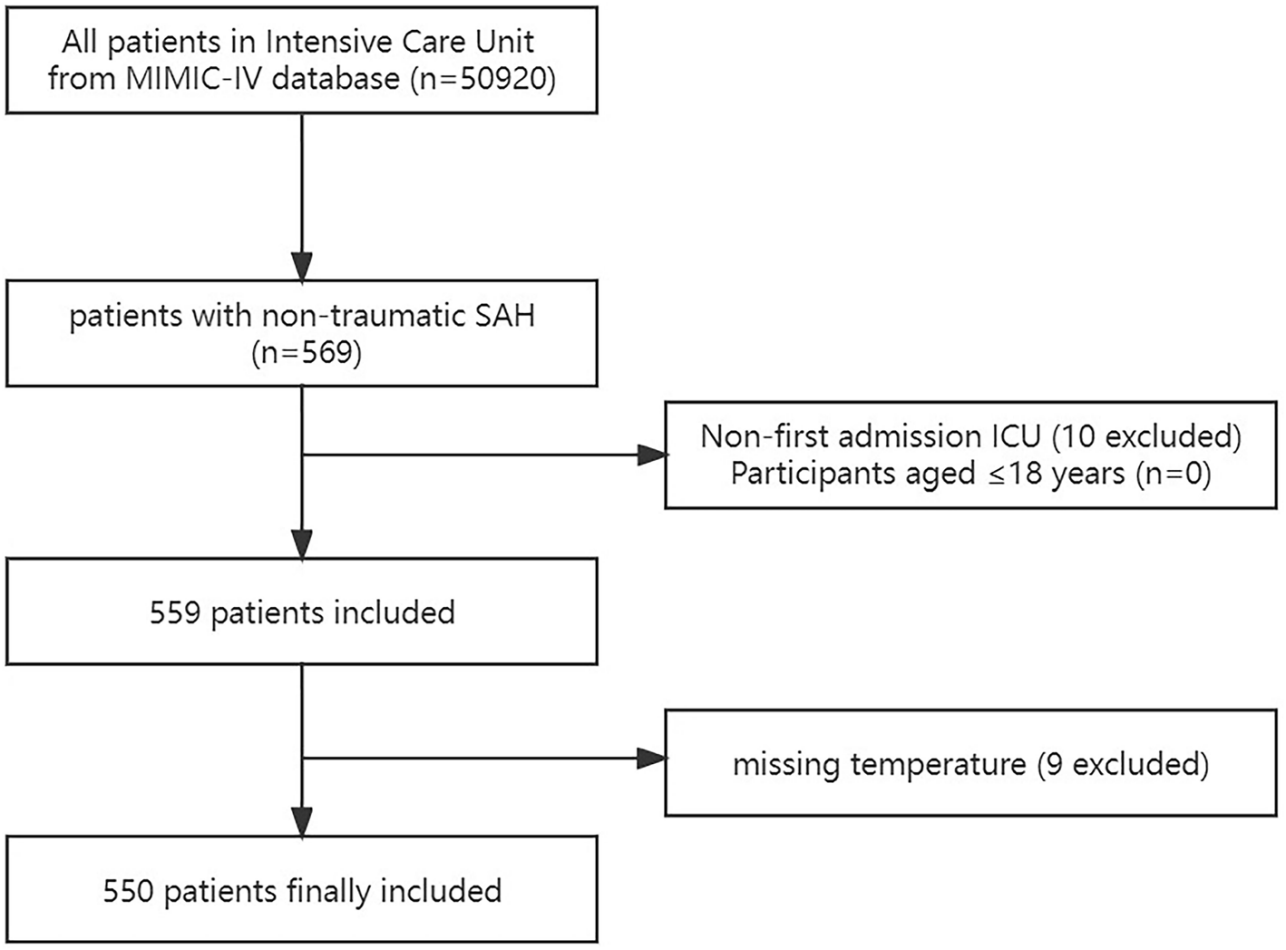
Flow chart of the study. MIMC-IV, Medical Information Mart for Intensive Care IV; ICU, intensive care unit; SAH, subarachnoid hemorrhage.

550 out of the 559 non-traumatic SAH patients that were assessed and ultimately included in the study were those who first had an ICU admission and satisfied the inclusion criteria (Fig. 1). Q1:31.30–36.06 °C; Q2:36.10–36.50 °C; Q3:36.56–36.72 °C; Q4:36.72–38.22 °C was the quartile used for dividing the T-lowest into four groups. Table 1 lists the patient characteristics for each group. Of the patients, 56.7% were female and had an average age of 61.1 ± 14.7 years. The patient’s demographic data, vital signs, comorbidities, hematological detection indicators, surgical therapy, score, outcome, and other relevant details are all included in Table 1. Between-group differences were statistically significant for age, heart rate, RR, blood glucose, hemoglobin, diabetes, sepsis, and Charlson comorbidity index. Comparing group Q3 to group Q1, the mortality rate for patients was lower.

The non-survival group had a lower T-lowest compared with the survival group (36.1 ± 1.2 ℃ vs. 36.4 ± 0.6 ℃). In addition, the non-survival group had the characteristics of older age (68.5 ± 13.9 years vs. 58.8 ± 14.3 years), higher Charlson comorbidity index, and lower GCS score, as shown in Supplementary Table 2.

### Association of T-lowest with in-hospital mortality

Except the T-lowest, univariate analysis revealed that the following factors were correlated with in-hospital mortality: age, RR, heart rate, T-mean, SpO_2_, severe liver disease, Charlson comorbidity index, blood glucose, hemoglobin, platelet count, WBC, calcium, and whether intravascular therapy was employed. The association between T-lowest and in-hospital mortality is presented in Table 2 both unadjusted and multivariable adjusted. Model 1 had adjustments for age, race, and sex. Twelve factors were further adjusted in Model 2 based on Model 1, including heart rate, RR, SpO_2_, blood glucose, WBC, blood creatinine, endovascular treatment position, and GCS score. T-lowest was found to be correlated with mortality when it was used as a continuous variable (unadjusted model: HR = 0.61, 95%CI: 0.51–0.73, *p* < 0.001; Model I: HR = 0.66, 95%CI: 0.56–0.78, *p* < 0.001; Model II: HR = 0.72, 95%CI: 0.59–0.86, *p* < 0.001). Model II indicated that for every 1 °C increase in T-lowest, the in-hospital mortality dropped by 28%. Using T-lowest as the categorical variable, group Q3 (36.56–36.72 ℃) had an adjusted HR value of 0.5 (95%CI: 0.28–0.87, *p* = 0.014) compared to reference group Q1 (31.30–36.06 ℃), and the risk of in-hospital death was 50% lower than in group Q1. But the group Q2 (36.10–36.50 °C, HR: 0.77, 95% CI: 0.44–1.32, *p* = 0.339) and Q4 (36.72–38.22 °C, HR: 0.8, 95% CI: 0.51–1.25, *p* = 0.329) did not show significant statistical differences. The results of other Models based on a confounding variable screening method of DAG are consistent with Model II (Supplementary Table 7).

**Table 2:** Multivariate cox regression analyses for hospital mortality in non-traumatic subarachnoid hemorrhage patients.

Exposure	Non-adjust model		Model I		Model II	
	HR (95% CI)	*p*-value	HR (95% CI)	*p*-value	HR (95% CI)	*p*-value
T-lowest quartiles						
Q1(31.30–36.06 ^◦^C)	1 (Ref)		1 (Ref)		1 (Ref)	
Q2(36.10–36.50^**◦ **^C)	0.58 (0.35 ~ 0.94)	0.026	0.67 (0.41 ~ 1.1)	0.114	0.77 (0.44 ~ 1.32)	0.339
Q3(36.56–36.72 ^◦^C)	0.39 (0.23 ~ 0.67)	0.001	0.45 (0.26 ~ 0.77)	0.004	0.5 (0.28 ~ 0.87)	0.014
Q4(36.72–38.22 ^**◦**^C)	0.7 (0.46 ~ 1.08)	0.109	0.71 (0.46 ~ 1.11)	0.133	0.8 (0.51 ~ 1.25)	0.329
*p* for trend	0.87 (0.75 ~ 1.02)	0.078	0.88 (0.75 ~ 1.02)	0.087	0.91 (0.78 ~ 1.05)	0.198
T-lowest (per 1 increases)	0.61 (0.51 ~ 0.73)	< 0.001	0.66 (0.56 ~ 0.78)	< 0.001	0.72 (0.59 ~ 0.86)	< 0.001

Non-adjusted: no covariates were adjusted.

Model I: adjusted for age, sex, and ethnicity.

Model II: adjusted for age, sex, ethnicity, heart rate, RR, SpO_2_, blood glucose, WBC, Charlson comorbidity index, Cr, endovascular therapy, and GCS.

*T-lowest* the lowest body temperature on the first day of ICU admission, *RR* respiratory rate, *SpO*_*2*_ percutaneous oxygen saturation, *WBC* white blood cell, *Cr* Creatinine, *GCS* Glasgow coma score, *HR* hazard ratio, *CI* confidence interval, *Ref* reference.

**Figure 2:**
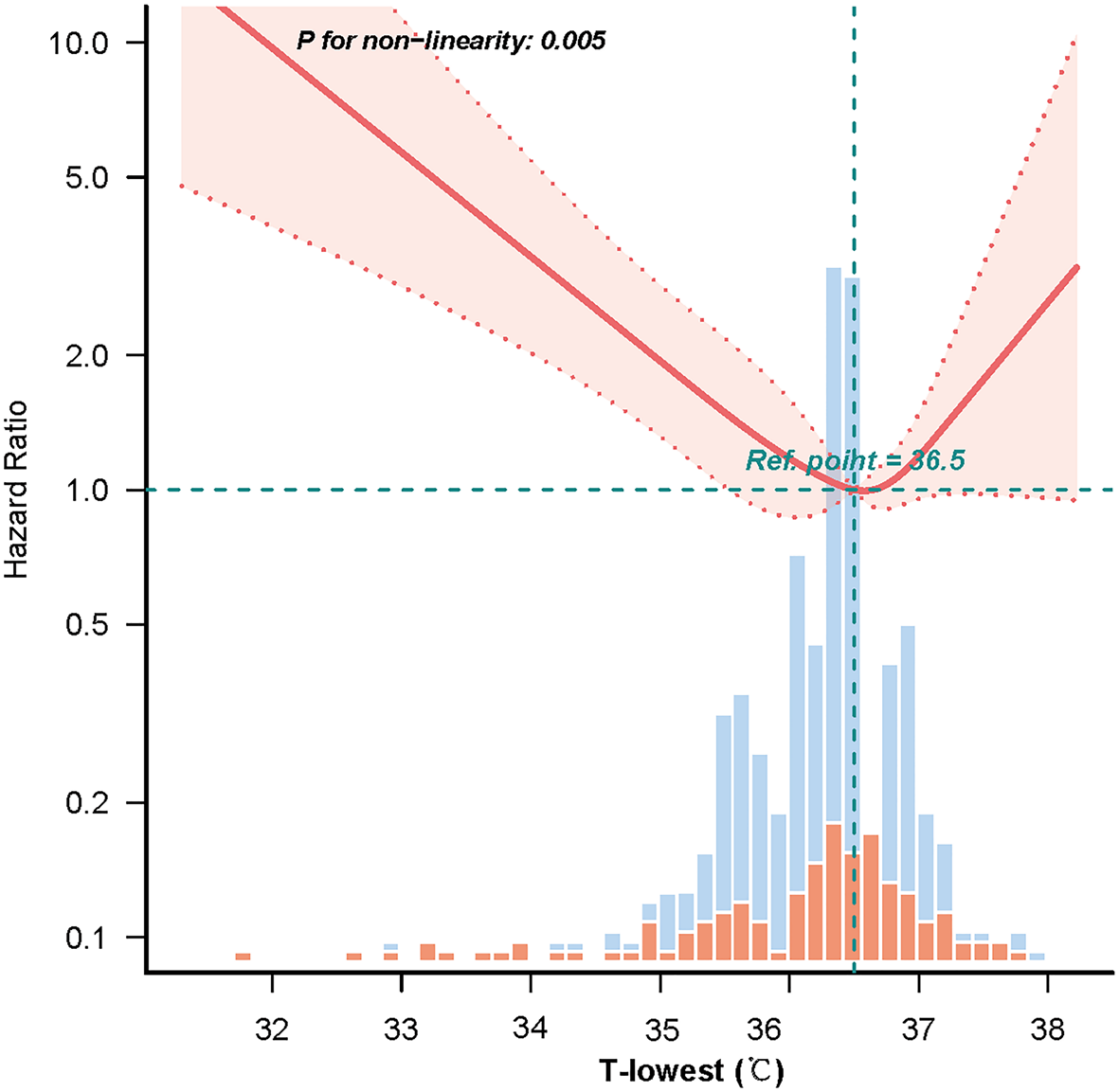
Restricted cubic spline plot for association between T-lowest and in-hospital mortality. The data were adjusted for the variables in Model II. T-lowest, the lowest body temperature on the first day of ICU admission.

**Figure 3:**
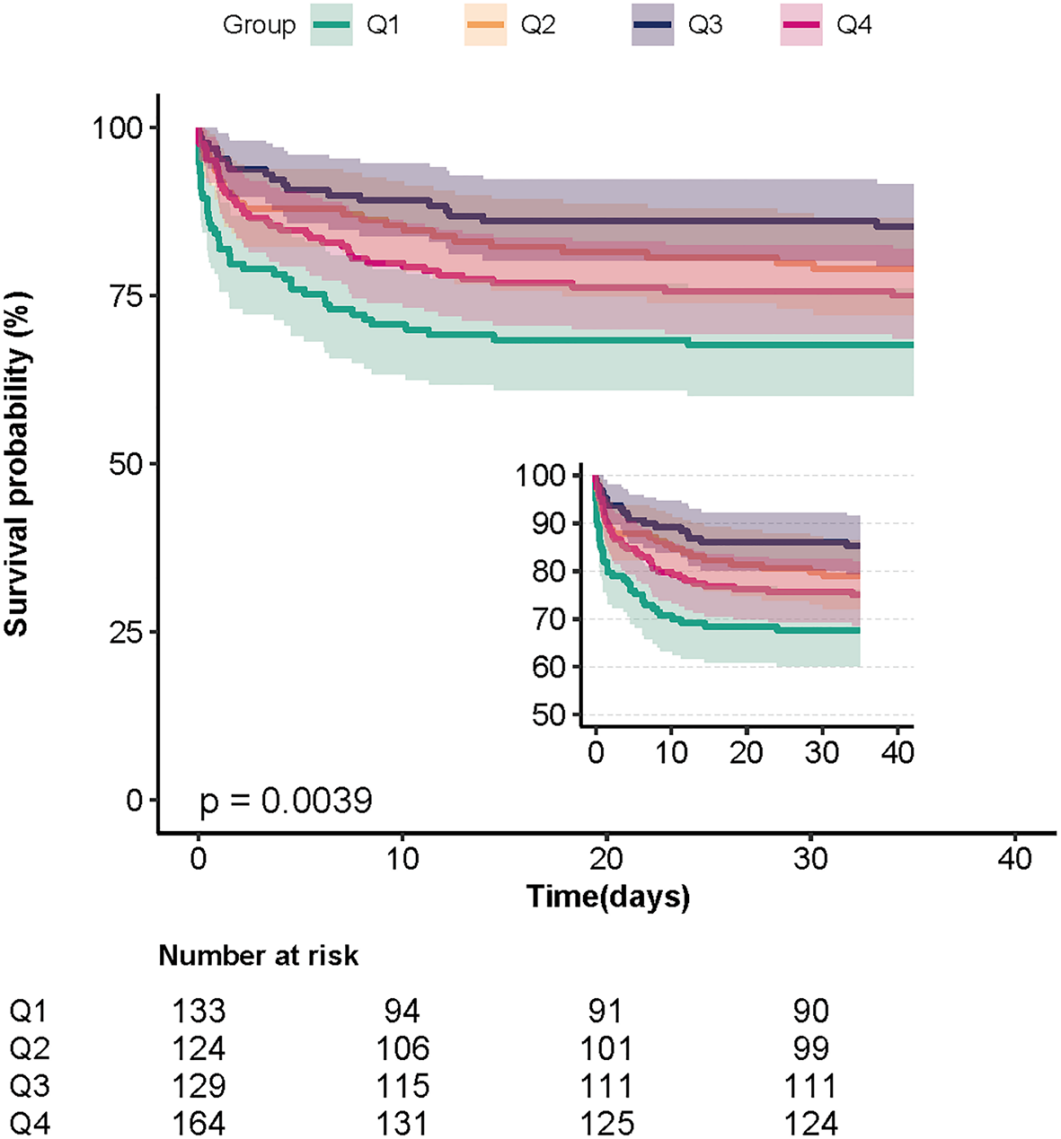
Kaplan–Meier survival curves for patients with non-traumatic SAH based on the T-lowest level. X-Axis: survival time (days). Y-Axis: survival probability. SAH, subarachnoid hemorrhage; T-lowest, the lowest body temperature on the first day of ICU admission.

Changes in HR values between the quartile groups suggest that the relationship between T-lowest and in-hospital mortality may be non-linear. Multivariate-adjusted restricted cubic spline analyses using covariates of Model II showed a non-linear relation association between T-lowest and in-hospital mortality (Fig. 2, *p* = 0.005). The mortality rate is lowest when the T-lowest is close to 36.5 °C, and the risk of death is increased when the temperature is lower or higher than that. Figure 3 displays the K-M curves between the four groups. The chart shows that group Q3’s survival rate was considerably greater than group Q1’s (*p* = 0.0039).

### Subgroup analysis

**Figure 4:**
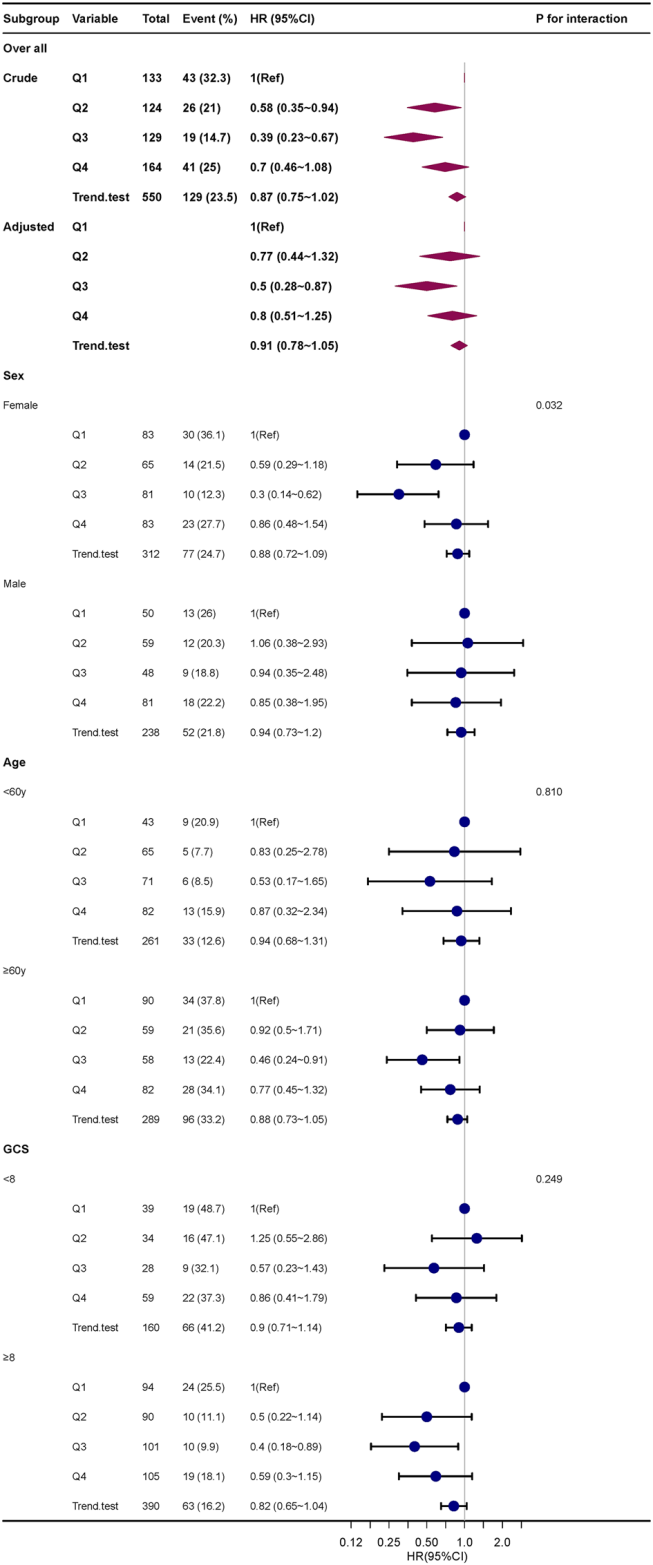
Subgroup analyses of the effect of in-hospital mortality. Adjusted for age, sex, ethnicity, heart rate, RR, SpO_2_, blood glucose, WBC, Charlson comorbidity index, Cr, endovascular therapy, and GCS. *RR* respiratory rate, *SpO*_*2*_ percutaneous oxygen saturation, *WBC* white blood cell, *Cr* creatinine, *GCS* glasgow coma score, *HR* hazard ratio, *CI* confidence interval, *Ref* reference.

Subgroup analysis revealed no significant interactions among different subgroups including age (< 60 years and ≥ 60 years), sex, and GCS (< 8 and ≥ 8) (Fig. 4). There was a stable association between T-lowest and in-hospital mortality.

### Sensitivity analysis

T-lowest was divided into group 1 (< 36℃) and group 2 (≥ 36℃) using 36℃ as the cut-off value. The analysis of Supplement Table 4 revealed that there was an increased likelihood of mortality in the hypothermia group (Model II: HR = 0.65, 95%CI: 0.44–0.97, *p* = 0.035). Supplementary Fig. 1 displays the K-M curve comparing the two groups. The survival rate of group 1 was substantially lower than that of group 2, as the figure shows (*p* < 0.001).

We also performed multivariate regression analyses of T-lowest levels and mortality at 24 h, 48 h, 7 days, 3 months, 6 months, and 1 year, and the results remained stable (Supplement Table 5). The K-M curves of mortality at these time points are shown in Supplementary Fig. 2.

This study also conducted a multivariate regression analysis on the relationship between T-mean and in-hospital mortality, and the results showed that in-hospital mortality was also negatively correlated with T-mean (supplement Table 6). The T-mean are grouped by quartile, and the K-M curves between the four groups are shown in Supplementary Fig. 3. Group Q1 had a considerably lower survival rate (*p* < 0.001) than the other groups, as the figure illustrates.

## Discussion

We studied the connection between T-lowest on the first day of ICU admission and in-hospital mortality in non-traumatic SAH patients. Our analysis showed that after accounting for other factors, T-lowest had a nonlinearly correlation with in-hospital mortality. This information can be beneficial in predicting outcomes for individuals with non-traumatic SAH who are admitted to the ICU.

Monitoring and management of body temperature is critical in the management of SAH patients admitted to the ICU. Most of the studies published so far have only focused on the effect of hyperthermia after SAH on prognosis, ignoring the relationship between baseline body temperature at admission and patient prognosis. However, the relationship between body temperature and brain physiology, and clinical outcomes is complex. Animal studies have shown that mild hypothermia (32–34 °C) has the potential to limit the degree of secondary brain injury after aneurysmal SAH by reversing cerebral perfusion, preventing cerebral edema formation, and reducing cerebral vasospasm^29–31^. Although the exact mechanism of the decrease in intracranial pressure associated with hypothermia remains to be elucidated, it has previously been established that increased cerebrovascular resistance leads to a decrease in cerebral blood flow (CBF), which leads to a decrease in intracranial pressure^32^. However, short-term hypothermia therapy during aneurysm surgery does not improve the prognosis of patients with severe SAH^33^. A lower body temperature (33 °C) is an important determinant of reduced blood flow velocity and may reflect lower CBF^34^. Under physiological conditions, hypothermia has been associated with reduced CBF^35^. This, in turn, may alter hemorheology, promote red blood cell aggregation, activate platelets and white blood cells, reduce microcirculatory blood flow, and ultimately lead to delayed cerebral infarction (DCI) after intracranial aneurysm rupture^34^. Other reports support this view. One study reported that hypothermia upon hospital admission was associated with early neurological deterioration in patients with lacunar infarction^36^. Another study reported that hypothermia within 6 h of onset was associated with severe neurological dysfunction in the early stages of cerebral infarction^37^. Other studies have also demonstrated that hypothermia therapy may not be beneficial for patients with malignant middle cerebral artery occlusion cerebral infarction, in part because of the higher incidence of adverse events in patients treated with hypothermia^38,39^. Importantly, DCI after the rupture of an intracranial aneurysm is a significant cause of mortality^40^. Hypothermia in the early stage of SAH may be attributed to a natural brain protective mechanism that is activated in the body shortly after SAH-induced transient global ischemic injury, or in part to changes in temperature set points due to toxic and inflammatory effects in the brain stem/hypothalamus and/or ischemic complications^41^. Although the low temperature has a protective effect on brain tissue, on the other hand, the lower the body temperature, the more complications such as arrhythmia, electrolyte disturbance, immunosuppression, and coagulation disorder will be caused^42,43^. Our research revealed that patients with SAH who had hypothermia on their first day of ICU treatment had a greater likelihood of mortality in the hospital. These findings highlight the need to keep the focus on the T-lowest in SAH patients and suggest that clinicians should consider the T-lowest level on the first day of hospitalization as an important reference for patient prognosis management.Upon doing a continuous variable analysis in this study, we discovered that, when T-lowest was grouped by quartile, there was no significant difference between group Q2 (36.10–36.50 ℃), group Q4 (36.72–38.22 ℃), and group Q1 (31.30–36.06 ℃). However, the risk of death was 50% lower in the Q3 group (36.56–36.72 ℃) compared to the Q1 group. Therefore, we attempted to explore whether there was a curved-line relationship between T-lowest and in-hospital mortality, and we found that the mortality rate is lowest when the T-lowest is close to 36.5 °C, and the risk of death is increased when the temperature is lower or higher than that. As the T-lowest rises, it is in line with clinical practice that the mortality rate does not continue to decrease. In addition, we performed sensitivity analyses to demonstrate the reliability of the main findings. T-lowest was divided into group 1 (< 36 ℃) and group 2 (≥ 36 ℃) using 36 ℃ as the cut-off value. The results showed that group 2 had a 35% lower risk of death than group 1. We also performed a multivariate regression analysis of T-lowest and multiple endpoints (24 h, 48 h, 7 day, 3 month, 6 month, and 1 year mortality), and the results showed that T-lowest was negatively correlated with mortality. To avoid temperature measurement errors, we also studied the T-mean of patient, and the results showed that in-hospital mortality was also negatively correlated with the T-mean. These results suggest that the slight change in body temperature on the first day of hospitalization in SAH patients may have a significant impact on their prognosis them.

Our study has the following advantages: (i) The relationship between T-lowest and in-hospital mortality among those suffering from non-traumatic SAH has not been reported in published papers to date; (ii) This study takes data from the real world to create a larger, more ethnically diversified population study. Lower null values for temperature and covariates included in multivariate regression analysis may reduce selection bias; (iii) According to the results of our study, SAH patients who had moderate T-lowest levels (For example, 36.56–36.72 ^◦^C) had a higher chance of surviving in the hospital. These findings point to the significance of T-lowest monitoring in SAH patients, which may aid doctors in identifying individuals at high risk for non-traumatic SAH and point to the need to pay more attention to patients whose temperature levels are dropping. (iv) We also performed several sensitivity analyses and found the results to be robust.

This study does have certain restrictions, though. First, retrospective observational designs inevitably have bias and confounding factors. Second, the results’ applicability to other groups of people may be constrained by this single-center sample. As a result, care should be used when interpreting and using our findings in different situations. Third, because of limitations in the MIMIC database, missing information that might have had an impact on the model, including the diagnosis of cerebral vasospasm, Fisher grading, was not acquired. Fourth, we analyzed first-day body temperature records collected during ICU hospitalization and did not analyze changes in body temperature during hospitalization, so the results were limited to a limited period. Questions regarding the duration of hypothermia during hospitalization should be evaluated with further research. However, a relationship between T-lowest on the first day of ICU admission and in-hospital mortality was revealed.

## Conclusion

Patients with low baseline temperature levels should thus get greater consideration since they may have a higher in-hospital mortality. This will allow clinicians to make better clinical decisions.

### Supplementary Information

Supplementary Information.

## Data Availability

In this study, data from public databases were analyzed. These data can be found here: MIMIC-IV: https://www.physionet.org/content/mimiciv/2.2/.
