# Supplementary material for: Hypothermia on the first day of ICU admission leads to increased in-hospital mortality in patients with subarachnoid hemorrhage
Source: Sci Rep. 2024 Apr 28;14:9730. doi: 10.1038/s41598-024-60657-8 (PMC11055887; doi:10.1038/s41598-024-60657-8)
Supplement: Supplementary file 1 — Supplementary Information. [file 41598_2024_60657_MOESM1_ESM.pdf]

Hypothermia on the first day of ICU admission leads to increased in-hospital mortality in patients with subarachnoid hemorrhage

Wenyuan Du[1*], Jingmian Yang[1], Yanfang Lou[1] and Jiahua You[1]

[1]Department of Neurology, Shijiazhuang Traditional Chinese Medicine Hospital, Shijiazhuang, Hebei, China

*Correspondence:
Wenyuan Du
wenyuan0831@163.com

Supplementary information includes:

(i) Supplementary Figures 1, 2, 3

(ii) Supplementary Tables 1, 2, 3, 4, 5, 6, 7

Supplementary Figure 1

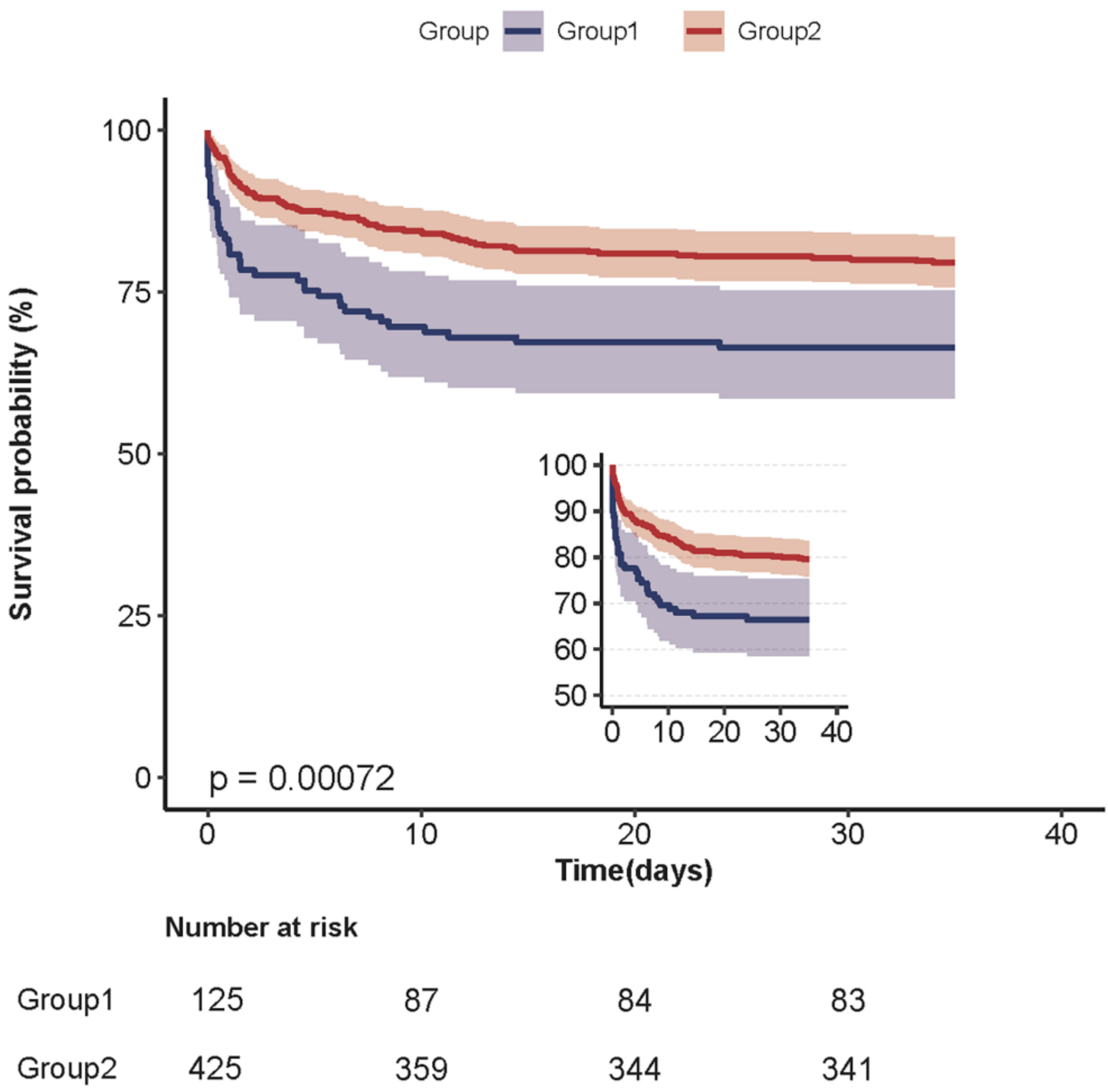

Figure S1 Kaplan–Meier survival curves for patients with non-traumatic SAH based on the T-lowest level. Group 1, T-lowest <36℃; Group 2, T-lowest≥36℃. T-lowest, the lowest body temperature on the first day of ICU admission.

Supplementary Figure 2

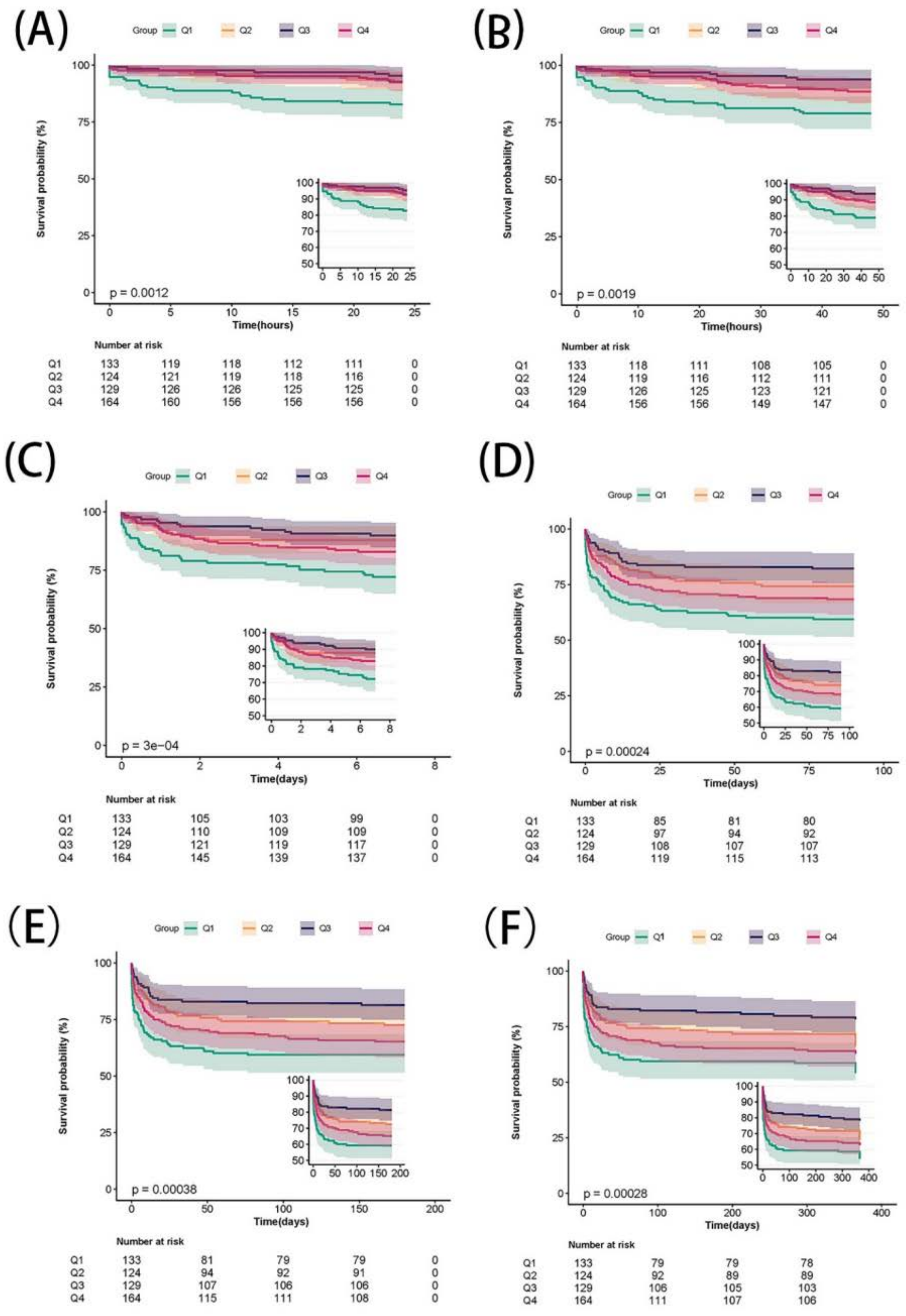

Figure S2 Kaplan–Meier survival curves based on the T-lowest level at different time points. (A) shows the 24-hour survival probability from different T-lowest groups. (B) shows the 48-hour survival probability from different T-lowest groups. (C) shows the 7-day survival probability from different T-lowest groups. (D) shows the 3-month survival probability from different T-lowest groups. (E) shows the 6-month survival probability from different T-lowest groups. (F) shows the 1-year survival probability from different T-lowest groups. X-Axis: survival time (hours/days). Y-Axis: survival probability. T-lowest, the lowest body temperature on the first day of ICU admission.

Supplementary Figure 3

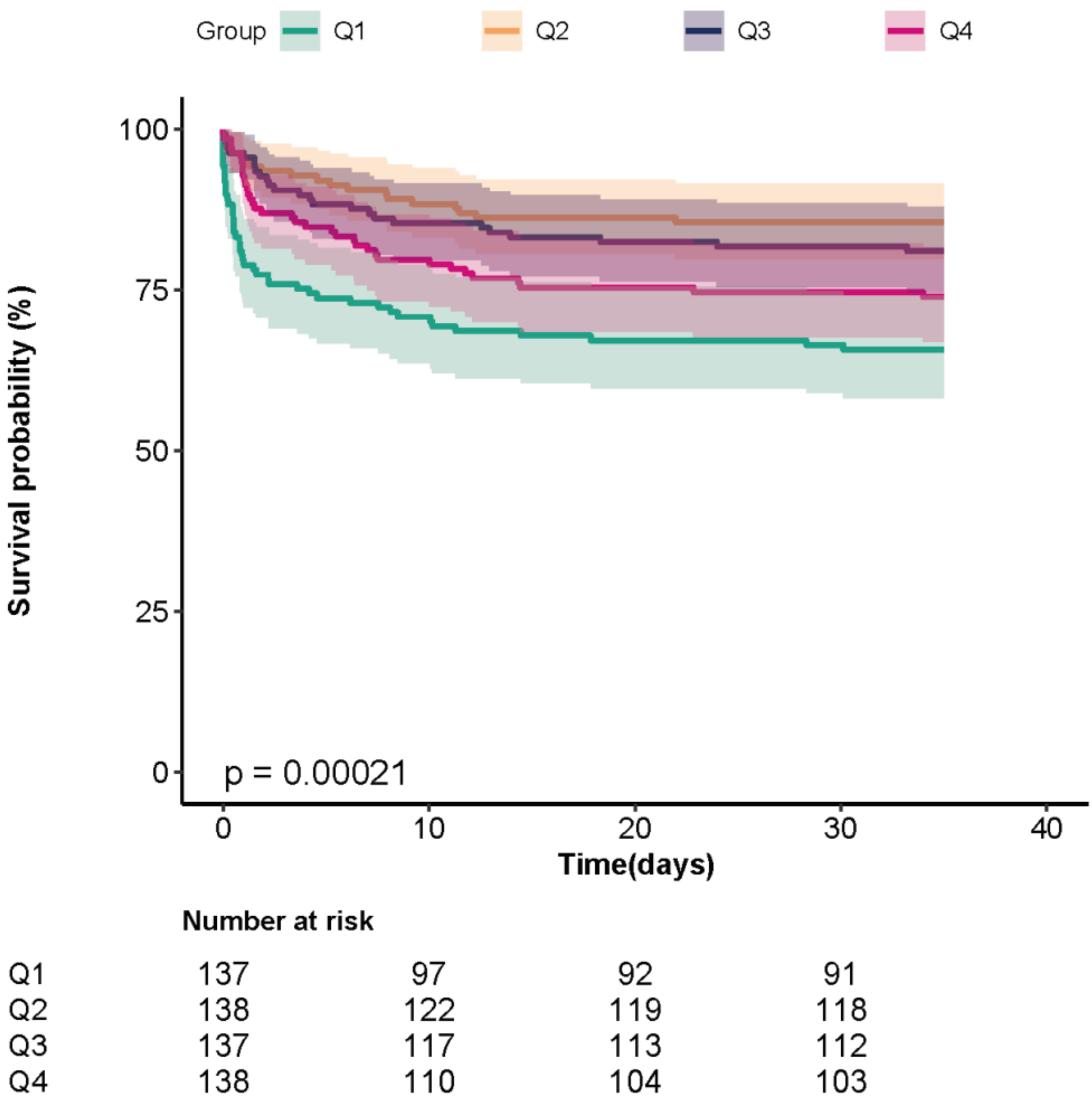

Figure S3 Kaplan–Meier survival curves based on the T-mean level. X-Axis: survival time (days). Y-Axis: survival probability. T-mean, the mean body temperature on the first day of ICU admission.

Supplementary T1 Details of missing values

Variable	Miss. freq	Miss. percentage%
Female	0	0
Age	0	0
Ethnicity	0	0
White	0	0
Black	0	0
Asian	0	0
Other	0	0
Heart rate	0	0
MBP	0	0
RR	0	0
SpO_2	0	0
Myocardial infarction	0	0
Congestive heart failure	0	0
Chronic pulmonary disease	0	0
Hypertension	0	0
Diabetes	0	0
Paraplegia	0	0
Sepsis	0	0
Renal disease	0	0
Malignant cancer	0	0
Severe liver disease	0	0
Charlson comorbidity index	0	0
Glucose	21	3.8182
RBC	37	6.7273
Hemoglobin	2	0.3636
Platelets	3	0.5455
WBC	2	0.3636
Sodium	14	2.5455
Calcium	33	6
PT	10	1.8182
APTT	15	2.7273
Cr	2	0.3636
BUN	2	0.3636
Endovascular therapy	0	0
Clipping of aneurysm	0	0
GCS	0	0
APSIII	0	0
SOFA	295	53.6364
Hydrocephalus	0	0
hospital mortality	0	0

MBP, mean blood pressure; RR, respiratory rate; SpO_2, percutaneous oxygen saturation; RBC, red blood cell; WBC, white blood cell; PT, prothrombin time; APTT, activated partial thromboplastin time; Cr, Creatinine; BUN,

Blood urea nitrogen; GCS, Glasgow coma score; APSIII score, Acute Physiology III score; SOFA, Sequential Organ Failure Assessment.

Supplementary T2 Population characteristics between different groups.

Variables	Group			p-value
	Total (n = 550)	Survivors (n = 421)	Non-survivors (n = 129)	
**Demographic**				
Female, n (%)	312 (56.7)	235 (55.8)	77 (59.7)	0.438
Age, years	61.1 ± 14.7	58.8 ± 14.3	68.5 ± 13.9	< 0.001
Ethnicity, n (%)				< 0.001
White	345 (62.7)	286 (67.9)	59 (45.7)	
Black	46 (8.4)	39 (9.3)	7 (5.4)	
Asian	18 (3.3)	10 (2.4)	8 (6.2)	
Other	141 (25.6)	86 (20.4)	55 (42.6)	
**Vital signs**				
Heart rate, beats/min	78.0 ± 13.8	76.8 ± 12.9	82.0 ± 15.6	< 0.001
MBP, mmHg	81.7 ± 9.0	81.8 ± 8.9	81.2 ± 9.5	0.488
RR, times/min	18.0 ± 3.3	17.5 ± 3.1	19.3 ± 3.8	< 0.001
T-lowest, ◦C	36.3 ± 0.8	36.4 ± 0.6	36.1 ± 1.2	< 0.001
Quartiles of T-lowest				0.008
Q1(31.30-36.06◦C)	133 (24.2)	90 (21.4)	43 (33.3)	
Q2(36.10-36.50◦C)	124 (22.5)	98 (23.3)	26 (20.2)	
Q3(36.56-36.72◦C)	129 (23.5)	110 (26.1)	19 (14.7)	
Q4(36.72-38.22◦C)	164 (29.8)	123 (29.2)	41 (31.8)	
T-mean, ◦C	37.0 ± 0.6	37.0 ± 0.4	36.8 ± 1.0	0.003
SpO_2, %	97.7 (96.0, 98.9)	97.6 (96.1, 98.9)	98.1 (95.5, 99.5)	0.270
**Comorbidities, n (%)**				
Myocardial infarction	41 (7.5)	29 (6.9)	12 (9.3)	0.361
Congestive heart failure	41 (7.5)	29 (6.9)	12 (9.3)	0.361
Chronic pulmonary disease	78 (14.2)	55 (13.1)	23 (17.8)	0.175
Hypertension	276 (50.2)	204 (48.5)	72 (55.8)	0.144
Diabetes	75 (13.6)	54 (12.8)	21 (16.3)	0.317
Paraplegia	64 (11.6)	47 (11.2)	17 (13.2)	0.532
Sepsis	255 (46.4)	187 (44.4)	68 (52.7)	0.098
Renal disease	30 (5.5)	19 (4.5)	11 (8.5)	0.079
Malignant cancer	22 (4.0)	17 (4)	5 (3.9)	0.935
Severe liver disease	7 (1.3)	3 (0.7)	4 (3.1)	0.056
Charlson comorbidity index	4.0 (3.0, 6.0)	4.0 (3.0, 6.0)	5.0 (4.0, 7.0)	< 0.001
**Laboratory results**				
Glucose, mg/dl	142.2 ± 37.0	136.4 ± 31.9	164.5 ± 45.8	< 0.001
RBC, 10^{12}/L	4.0 ± 0.7	4.0 ± 0.6	4.0 ± 0.8	0.876
Hemoglobin, g/L	11.9 ± 1.9	12.0 ± 1.8	11.5 ± 2.2	0.009
Platelets, 10^{9}/L	216.9 ± 87.9	222.3 ± 84.9	199.0 ± 95.5	0.009
WBC, 10^{9}/L	11.1 ± 4.9	10.6 ± 4.1	12.7 ± 6.6	< 0.001
Sodium, mmol/L	138.0 ± 3.9	138.1 ± 3.4	137.9 ± 5.4	0.650
Calcium, mmol/L	8.3 ± 0.7	8.4 ± 0.7	8.2 ± 0.8	0.012
PT, s	12.2 (11.3, 13.0)	12.1 (11.2, 12.9)	12.4 (11.4, 13.6)	0.032

APTT, s	26.2 (23.7, 28.5)	26.1 (23.7, 28.4)	26.2 (23.7, 29.5)	0.685
Cr (mg/dL)	0.7 (0.6, 0.9)	0.7 (0.6, 0.9)	0.8 (0.6, 1.1)	< 0.001
BUN (mg/dL)	13.0 (10.0, 17.0)	12.0 (9.0, 15.2)	16.0 (12.8, 24.0)	< 0.001
**Therapy and outcome, n (%)**				
Endovascular therapy	195 (35.5)	168 (39.9)	27 (20.9)	< 0.001
Clipping of aneurysm	38 (6.9)	33 (7.8)	5 (3.9)	0.121
Hydrocephalus	163 (29.6)	118 (28)	45 (34.9)	0.136
**Scores**				
GCS	13.0 (7.0, 14.0)	13.0 (8.0, 14.0)	7.0 (3.0, 15.0)	0.015
APSIII	38.5 (27.0, 60.0)	34.0 (26.0, 51.0)	61.0 (44.0, 88.0)	< 0.001
SOFA	3.0 (2.0, 4.0)	2.0 (2.0, 3.0)	3.0 (2.0, 4.2)	0.011

MBP, mean blood pressure; RR, respiratory rate; T-lowest, the lowest body temperature on the first day of ICU admission; T-mean, the mean body temperature on the first day of ICU admission; SpO_2, percutaneous oxygen saturation; RBC, red blood cell; WBC, white blood cell; PT, prothrombin time; APTT, activated partial thromboplastin time; Cr, Creatinine; BUN, Blood urea nitrogen; GCS, Glasgow coma score; APSIII score, Acute Physiology III score; SOFA, Sequential Organ Failure Assessment.

Supplementary T3 Univariate analysis

Item	HR(95%CI)	*p*-value
Sex		
Female	Ref.	
Male	0.86 (0.61,1.23)	0.411
Age	1.04 (1.03,1.06)	< 0.001
Ethnicity		
White	Ref.	
Black	0.89 (0.41,1.94)	0.767
Asian	3.18 (1.52,6.66)	0.002
Other	2.59 (1.79,3.73)	< 0.001
Heart rate	1.02 (1.01,1.04)	< 0.001
MBP	0.99 (0.97,1.01)	0.469
RR	1.13 (1.08,1.18)	< 0.001
T-lowest	0.61 (0.51,0.73)	< 0.001
Quartiles of T-lowest		
Q1(31.30-36.06°C)	Ref.	
Q2(36.10-36.50°C)	0.58 (0.35,0.94)	0.026
Q3(36.56-36.72°C)	0.39 (0.23,0.67)	< 0.001
Q4(36.72-38.22°C)	0.7 (0.46,1.08)	0.109
T-mean	0.6 (0.48,0.76)	< 0.001
SpO_2	0.85 (0.8,0.91)	< 0.001
Myocardial infarction		
No	Ref.	
Yes	1.27 (0.7,2.29)	0.435
Congestive heart failure		
No	Ref.	
Yes	1.33 (0.73,2.41)	0.348
Chronic pulmonary disease		
No	Ref.	
Yes	1.31 (0.84,2.06)	0.237
Hypertension		
No	Ref.	
Yes	1.28 (0.9,1.81)	0.163
Diabetes		
No	Ref.	
Yes	1.27 (0.8,2.03)	0.31
Paraplegia		
No	Ref.	
Yes	1.14 (0.68,1.9)	0.618
Sepsis		
No	Ref.	
Yes	1.24 (0.88,1.76)	0.217
Renal disease		

No	Ref.	
Yes	1.76 (0.95,3.27)	0.073
Malignant cancer		
No	Ref.	
Yes	0.9 (0.37,2.19)	0.809
Severe liver disease		
No	Ref.	
Yes	2.88 (1.06,7.79)	0.037
Charlson comorbidity index	1.17 (1.1,1.25)	< 0.001
Glucose	1.01 (1.01,1.02)	< 0.001
RBC	1.05 (0.77,1.42)	0.757
Hemoglobin	0.89 (0.82,0.98)	0.013
Platelets	0.997 (0.995,0.999)	0.01
WBC	1.07 (1.04,1.1)	< 0.001
Sodium	0.995 (0.948,1.045)	0.836
Calcium	0.74 (0.58,0.94)	0.013
PT	1.06 (1.02,1.1)	0.003
APTT	1.00 (0.99,1.02)	0.693
Cr	1.4 (1.22,1.62)	< 0.001
BUN	1.03 (1.02,1.03)	< 0.001
Endovascular therapy		
No	Ref.	
Yes	0.42 (0.28,0.65)	< 0.001
Clipping of aneurysm		
No	Ref.	
Yes	0.48 (0.2,1.18)	0.112
GCS	0.91 (0.87,0.94)	< 0.001
APSIII	1.03 (1.03,1.04)	< 0.001
SOFA	1.15 (1.03,1.29)	0.013
Hydrocephalus		
No	Ref.	
Yes	1.29 (0.9,1.85)	0.171

MBP, mean blood pressure; RR, respiratory rate; T-lowest, the lowest body temperature on the first day of ICU admission; T-mean, the mean body temperature on the first day of ICU admission; SpO_2, percutaneous oxygen saturation; RBC, red blood cell; WBC, white blood cell; PT, prothrombin time; APTT, activated partial thromboplastin time; Cr, Creatinine; BUN, Blood urea nitrogen; GCS, Glasgow coma score; APSIII score, Acute Physiology III score; SOFA, Sequential Organ Failure Assessment.

Supplementary T4 Multivariate cox regression analyses for hospital mortality.

Exposure	Non-adjust model		Model I		Model II	
	HR (95% CI)	*p*-value	HR (95% CI)	*p*-value	HR (95% CI)	*p*-value
Groups of T-lowest						
Group1(<36)	1(Ref)		1(Ref)		1(Ref)	
Group2(≥36)	0.53 (0.37~0.77)	0.001	0.59 (0.41~0.87)	0.007	0.65 (0.44~0.97)	0.035

Non-adjusted: no covariates were adjusted.

Model I: adjusted for age, sex, and ethnicity.

Model II: adjusted for age, sex, ethnicity, heart rate, RR, SpO_2, blood glucose, WBC, Charlson comorbidity index, Cr, endovascular therapy, and GCS.

T-lowest, the lowest body temperature on the first day of ICU admission; RR, respiratory rate; SpO_2, percutaneous oxygen saturation; WBC, white blood cell; Cr, Creatinine; GCS, Glasgow coma score; HR, hazard ratio; CI, confidence interval; Ref, reference.

Supplementary T5 Multivariate cox regression analyses for different outcomes.

Exposure	Outcome	Non-adjust model		Model I		Model II	
		**HR (95% CI)**	***p*-value**	**HR (95% CI)**	***p*-value**	**HR (95% CI)**	***p*-value**
T-lowest (per 1 increases)	24h mortality	0.52 (0.42~0.63)	<0.001	0.55 (0.44~0.68)	<0.001	0.51 (0.39~0.66)	<0.001
	48h mortality	0.54 (0.45~0.65)	<0.001	0.58 (0.48~0.7)	<0.001	0.58 (0.46~0.73)	<0.001
	7d mortality	0.56 (0.47~0.66)	<0.001	0.61 (0.51~0.72)	<0.001	0.66 (0.54~0.8)	<0.001
	3m mortality	0.62 (0.52~0.74)	<0.001	0.68 (0.58~0.8)	<0.001	0.72 (0.61~0.86)	<0.001
	6m mortality	0.64 (0.54~0.77)	<0.001	0.7 (0.59~0.83)	<0.001	0.73 (0.62~0.87)	<0.001
	1y mortality	0.63 (0.54~0.75)	<0.001	0.69 (0.59~0.81)	<0.001	0.72 (0.61~0.85)	<0.001

Non-adjusted: no covariates were adjusted.

Model I: adjusted for age, sex, and ethnicity.

Model II: adjusted for age, sex, ethnicity, heart rate, RR, SpO_2, blood glucose, WBC, Charlson comorbidity index, Cr, endovascular therapy, and GCS.

T-lowest, the lowest body temperature on the first day of ICU admission; 24h, 24-hour; 48h, 48-hour; 7d, 7-day; 3m, 3-month; 6m, 6-month; 1y, 1-year; RR, respiratory rate; SpO_2, percutaneous oxygen saturation; WBC, white blood cell; Cr, Creatinine; GCS, Glasgow coma score; HR, hazard ratio; CI, confidence interval; Ref, reference.

Supplementary T6 Multivariate cox regression analyses for hospital mortality.

Exposure	Non-adjust model		Model I		Model II	
	HR (95% CI)	*p*-value	HR (95% CI)	*p*-value	HR (95% CI)	*p*-value
**T-mean quartiles**						
Q1(32.23-36.685)	1(Ref)		1(Ref)		1(Ref)	
Q2(36.686-36.956)	0.36 (0.21~0.6)	<0.001	0.42 (0.25~0.72)	0.002	0.4 (0.23~0.69)	0.001
Q3(36.96-37.28)	0.47 (0.29~0.77)	0.002	0.43 (0.26~0.7)	0.001	0.35 (0.21~0.59)	<0.001
Q4(37.284-38.96)	0.68 (0.44~1.05)	0.083	0.7 (0.45~1.1)	0.124	0.65 (0.4~1.05)	0.08
p for trend	0.89 (0.76~1.04)	0.138	0.87 (0.74~1.02)	0.092	0.83 (0.7~0.99)	0.036
**T-mean (per 1 increases)**	0.6 (0.48~0.76)	<0.001	0.65 (0.53~0.81)	<0.001	0.67 (0.54~0.82)	<0.001

Non-adjusted: no covariates were adjusted.

Model I: adjusted for age, sex, and ethnicity.

Model II: adjusted for age, sex, ethnicity, heart rate, RR, SpO_2, blood glucose, WBC, Charlson comorbidity index, Cr, endovascular therapy, and GCS.

T-mean, the mean body temperature on the first day of ICU admission; RR, respiratory rate; SpO_2, percutaneous oxygen saturation; WBC, white blood cell; Cr, Creatinine; GCS, Glasgow coma score; HR, hazard ratio; CI, confidence interval; Ref, reference.

Supplementary T7 Multivariate cox regression analyses for in-hospital mortality in non-traumatic subarachnoid hemorrhage patients.

**Exposure**	**Model III**		**Model IV**		**Model V**		**Model VI**		**Model VII**	
	**HR (95% CI)**	***p*-value**	**HR (95% CI)**	***p*-value**	**HR (95% CI)**	***p*-value**	**HR (95% CI)**	***p*-value**	**HR (95% CI)**	***p*-value**
T-lowest quartiles										
Q1(31.30-36.06)	1 (Ref)		1 (Ref)		1 (Ref)		1 (Ref)		1 (Ref)	
Q2(36.10-36.50)	0.78 (0.48~1.29)	0.337	0.78 (0.48~1.29)	0.339	0.77 (0.47~1.26)	0.296	0.74 (0.45~1.22)	0.241	0.75 (0.45~1.23)	0.256
Q3(36.56-36.72)	0.51 (0.3~0.89)	0.018	0.51 (0.3~0.89)	0.018	0.5 (0.29~0.88)	0.015	0.48 (0.28~0.84)	0.01	0.47 (0.27~0.81)	0.007
Q4(36.72-38.22)	0.76 (0.49~1.17)	0.213	0.76 (0.49~1.17)	0.216	0.75 (0.49~1.16)	0.203	0.74 (0.48~1.14)	0.168	0.76 (0.49~1.18)	0.22
p for trend	0.89 (0.77~1.04)	0.136	0.89 (0.77~1.04)	0.138	0.89 (0.77~1.03)	0.132	0.89 (0.76~1.03)	0.109	0.89 (0.77~1.03)	0.13
T-lowest (per 1 increases)	0.72 (0.6~0.85)	<0.001	0.72 (0.6~0.85)	<0.001	0.71 (0.6~0.85)	<0.001	0.71 (0.6~0.84)	<0.001	0.71 (0.6~0.85)	<0.001

Model III: adjusted for age, endovascular therapy, and GCS.

Model IV: adjusted for age, endovascular therapy, GCS and Charlson comorbidity index.

Model V: adjusted for age, endovascular therapy, GCS, Charlson comorbidity index and sepsis.

Model VI: adjusted for age, endovascular therapy, GCS, Charlson comorbidity index, sepsis, and clipping.

Model VII: adjusted for age, endovascular therapy, GCS, Charlson comorbidity index, sepsis, clipping and sex.

T-lowest, the lowest body temperature on the first day of ICU admission; GCS, Glasgow coma score; CI, confidence interval; Ref, reference.
